# Microencapsulation of Omeprazole by* Lactobacillus acidophilus* ATCC 4356 Surface Layer Protein and Evaluation of its Stability in Acidic Condition

**DOI:** 10.22037/ijpr.2019.111681.13306

**Published:** 2020

**Authors:** Elahe Mobarak Qamsari, Rouha Kasra Kermanshahi, Mohammad Erfan, Parinaz Ghadam

**Affiliations:** a *Department of Microbiology, Faculty of Biological Sciences, Alzahra University, Tehran, Iran. *; b *Department of Pharmaceutics, School of Pharmacy, Shahid Beheshti University of Medical Sciences, Tehran, Iran. *; c *Department of Biotechnology, Faculty of Biological Sciences, Alzahra University, Tehran, Iran. *

**Keywords:** Encapsulation, Lactobacillus, Omeprazole, Surface layer protein, Stability

## Abstract

The present study introduces a novel method for encapsulation of the acid-labile drug called Omeprazole using *Lactobacillus acidophilus (L. acidophilus) *ATCC 4356 S-layer protein. Before preparing the Omeprazole suspension, a series of preliminary studies were performed on the Omeprazole powder. For this purpose, some parameters such as melting point, IR spectrum, UV spectrum, and the particle size of Omeprazole powder were investigated. The size reduction process was done in order to achieve an ideal formulation. Ultimately, the resulting powder had an average particle size of 35.516 μm and it was almost uniform. After calculating the amount of S-layer protein required for complete covering of drug particles, the effect of different factors on the drug coating process was investigated with one factor at a time method. Then stability of coated Omeprazole was evaluated in acetate buffer (pH 5). Finally, the maximum coat of drug particles was determined using S- layer protein of *Lactobacillus acidophilus *ATCC 4356 at 25 °C for 2 h, shaking rate of 100 rpm and ratio of 2:1 for S-layer protein amount/Omeprazole Surface in Tris hydrochloride buffer medium (50 mM, pH 8). The coating of Omeprazole by the S-layer protein decreased the drug decomposition rate up to 2.223.

## Introduction

Surface layer (S-layer) protein is a crystalline structure of the cell envelope in some Bacteria and Archaea. The protein covers the cell surface and acts as protective coat maintaining the cell shape, trapping molecules and ions and participating in direct cell division, cell adhesion and surface recognition (1). The molecular mass of S-layer proteins ranges from approximately 40-200 kDa and they consist of arrays with oblique, square or hexagonal symmetric with pores ranging from 2 nm to 8 nm. Moreover, the thickness of S-layer structure varies from 5 nm to 20 nm (2). The monomeric units of S-layer protein interact with each other through non-covalent interactions. The most interesting feature of S-layer protein is its ability to reassemble after its disintegration. After treatment with chemical agents, the S-layer monomers can spontaneously reassemble to S-layer structures with the authentic symmetry pattern in different media such as solution, solid supports, lipid membranes and the air–water interface (3). S-layers demonstrate capability to possess a great potential in biotechnological and biomedical applications because of their self-assembling ability and highly ordered, regular structure at the nanometer scale (4, 5).

Microencapsulation is a process in which fine particles, liquid droplets or even gases are surrounded by a polymeric material. The obtained product is called microcapsule with diameter ranges of 1-1000 microns (6). Microencapsulation technology has become increasingly important in the development of pharmaceutical formulation and oral delivery systems (7, 8). Since the oral route is a simple, safe, non-invasive and repeatable method, it is more appropriate than other methods. For oral release applications, microencapsulation is commonly used with an insoluble polymer in water or with a hydrophilic polymer that liberates the drug through a gradual release mechanism (8).

A new idea in the field of microencapsulation is using lactobacillus S-layer nanostructures as the capsule coatings. Regarding the unique properties of the S-layer, especially the ability to self-assembling in different environments and the resistance of the bacteria possessing this layer against the gastric acid conditions, digestive enzymes and bile salts, this study aimed to use S-layer nanostructures of probiotic bacteria in order to coat the gastric acid susceptible drug (9). Recently, some studies have been conducted on S-layer proteins of* Lactobacillus kefir (L. kefir) *and *Lactobacillus brevis (L. brevis) *as protective coating of liposomes (as carriers of the vaccine) by oral route. The results showed that *L. kefir* and *L. brevis* S-layer proteins can adsorb positive surface liposome so that their sustainability in the digestive tract is increased (10-12).

Omeprazole belongs to the proton pump inhibitor (PPI) and it is one of the most important and highly digestible drugs. It acts through non-competitive inhibition of H^+^/K^+^- ATPase parietal cell. Omeprazole is the first clinically available and effective PPI drug used in the treatment of various acid-related diseases such as peptic ulcer, gastro-esophageal reflux diseases and Zollinger-Ellison syndrome so that it has been widely used for more than two decades (13). Omeprazole seems to be the well adsorbed from the gastrointestinal tract (GIT). However, its bioavailability following oral administration is usually very low (about 40% to 50%). The main problem with the production of Omeprazole suspension or oral solution is the instability and degradation of Omeprazole against gastric acid. Therefore, Omeprazole cannot be directly introduced into the acidic stomach (14, 15). Most of the current commercial Omeprazole products such as enteric-coated tablets and granules are delayed-release products (16). 

The purpose of the present study was to develop a novel Omeprazole encapsulation using S-layer nanostructures of *Lactobacillus acidophilus (L. acidophillus)* ATCC 4356 and to find optimal parameters for a stable Omeprazole formulation in acidic condition. 

## Experimental

*Bacterial Strain, growth condition and extraction of S-layer protein*

*L.acidophillus* ATCC 4356 was obtained from Iranian Research Organization for Science and Technology and propagated in de Man Rogosa Sharpe (MRS) broth under anaerobic condition at 37°C. For extraction of S-layer protein, the bacteria were harvested at the end of log phase by centrifugation (at 15000 ×g for 15 min at 4 °C) and washed twice with chilled distilled water. The cell pellets at the end of log phase were treated with 4M guanidine hydrochloride (GuHCL) in 50 mM Tris-HCl buffer (pH 7.2), (1 g of harvested cell pellets was suspended in 10-15 mL of 4M GuHCl) for 1 h at 37 °C. The extracted S-layer protein was separated from the cell pellets by centrifugation (18000 ×g, 15 min, 4 °C). The supernatant containing the S-layer protein was dialyzed over night at 4 °C against two liters of 50 mM Tris buffer (pH 7.2) with three time exchange of medium to remove residual GuHCl (17, 18).

*Determining the characteristics of Omeprazole*

Omeprazole powder was purchased from Temad pharmaceutical company of Iran. To ensure the purity of the drug, some of its properties such as melting point, IR spectrum and the UV spectrum have been investigated and the results were matched with Clark Reference (19). Since Omeprazole is insoluble in water, it should have a good particle size. Also, the uniformity of particle size results in better dispersion. Shredding of primary Omeprazole powder was done after two steps using a laboratory Ball Mill machine (60 min, 100 rpm). Shredded powder was collected using sieves in different mesh sizes (170, 200, 230, 270, and 400) (20). The particle size of obtained Omeprazole powder was measured using the Master Seizer device. 

*Preparation of Omeprazole suspension*

The approximate amount of S-layer protein required to cover the surface of drug was estimated based on the amount of adsorption of S-layer protein obtained from Quartz Crystal Microbalance (QCM) analysis and amount of drug specific surface area (21). According to calculations and the concentration of S-layer protein, an appropriate volume of S-layer protein was added to drug suspension with 1 mg/mL concentration. Total volume of the reaction was 5 mL in the Tris hydrochloride buffer medium (50 mM, pH 8) (22).

*Detection of drug coating by determining changes in protein concentration*

The amount of S-layer protein can be measured by the Bradford method (23). The initial amount of needed S-layer protein was obtained according to the calculations. Thus, by calculating the changes in the amount of S-layer protein at the end of reaction, the percentage of drug coating can be achieved. 

*Detection of drug coating through Omeprazole stability studies*

It is expected that if the drug is coated with S-layer protein, the stability of the drug will be increased in acidic pH (24). Therefore, the stability of Omeprazole coated with S-layer protein was investigated in pH 5 using the HPLC method. For this purpose, 1 mL of the coated drug was added to 9 mL of acetate buffer (pH 5). The solutions were stored at 30 ºC in incubator. At different time intervals of 0, 1, 2, 3 and 4 h the samples were taken out and were immediately frozen by Liquid nitrogen and were kept in a freezer (at -20 ºC) until they were analyzed. 

*HPLC method for Omeprazole assay*

HPLC method was used to determine the amount and the stability of Omeprazole. For this purpose, the monograph of oral suspension of Omeprazole in the United States Pharmacopeia (USP) was used (25). According to USP, the HPLC system was isocratically operated using a mobile phase, consisting of phosphate buffer with pH 8.5 and acetonitrile (65:35 v/v), previously degassed at a ﬂow rate of 1 mL/min. The column was C18 (3.9 mm × 15 cm, 5 μm particle size) equipped with an oven at 35 °C. The peak areas were automatically investigated using chromgate software and the results were expressed in terms of mean ± SD. A 1000 µg/mL stock Omeprazole solution was prepared in mobile phase. Standard Omeprazole solutions (10-100 µg/mL) were obtained with serial dilutions. Then standard calibration curve was plotted over the concentration range of 10-100 µg/mL. To evaluate the intra-day (repeatability) and inter-day (reproducibility) precision, three replicates of standard solutions at the range of calibration curve were assayed on the same day and on 3 different days (20). 

*Methods of drug coating using S-layer protein*

At the end of the dialysis process, the extracted S-layer proteins produce a polymeric structure. In most studies done on S-layer protein adsorption from different types of bacteria, protein monomers have been used (3, 26). Therefore, in this study, the main basis of the process was based on the use of extracted S-layer protein monomers and two methods have been used for drug coating process as the following. 

*Coating the drug during the dialysis process*

The onset of protein clouding in the dialysis bag indicated the maximum removal of the chemical extractant of the protein which is here GuHCl. This time was considered as the appropriate time to add drug. The dialysis process started with 4 mL of S-layer protein against the Tris buffer (50 mM, pH 8.5). Then, at the beginning of the third hour when the protein sample began to cloud, 1 mL of drug suspension was added to the dialysis bag. After 4 h, dialysis process was stopped and 1 mL of the product in the dialysis bag was added to 9 mL of acetate buffer (pH 5) and the stability of the coated drug was studied. 

*Coating the drug using S-layer protein monomers resulting from dialysis process*

After the end of dialysis process, the extracted protein was centrifuged (61000 ×g, 30 min, 4 °C). Transparent zone containing protein monomers is determined by the Bradford method and it was used to coat the drug. According to calculation and the concentration of S-layer protein, an appropriate volume of S-layer protein was added to the drug suspension with 1 mg/mL concentration. Total volume of reaction was 5 mL. The coating process was performed at 25 °C, 2 h, 100 rpm and (surface protein surface area)/(drug area) ratio of 2 in the Tris hydrochloride buffer medium (50 mM, pH 8) (22). Then, 1 mL of the product was added to 9 mL of acetate buffer (pH 5) and the stability of the coated drug was studied. It should be noted that in all experiments, falcon tubes were placed in incubator in a 45 degree angle in order to prevent sedimentation of drug particle as well as to provide more contact with S-layer proteins. To verify the repeatability, the coating process was performed using S-layer protein monomers under optimal conditions in 6 replicates and then CV (Coefficient of Variance) was calculated. 

*Optimization of Omeprazole coating by S-layer protein*

The experimental factors and their associated levels for Omeprazole coating via one factor at a time experiments are shown in Table 1. All experiments were performed in triplicates and the results were reported as the mean of these replications. 

*Statistics analysis*

Data was obtained by mean ± SD. For statistical analysis of numbers, SPSS V.21 software, independent sample *t*-test and nonlinear regression with Boolean algebra were used. The significance level of the differences with *p-*value less than 0.05 was calculated.

It should be noted that because of the fluidity of the reaction medium and also due to the equilibrium reaction of the binding of S-layer monomer to the surface, the protective effect of protein on the drug does not follow a regular linear pattern at different times. Therefore, to investigate the protective effect of S-layer protein on the drug, the significance of the difference in Y intercept at the zero point and the comparison of slope between two treatment and control samples at different times were investigated by nonlinear regression analysis. The Y intercept is the value of Y for X = 0. The slope of a line shows the change in Y values versus changes in X values at each point (27).

## Results and Discussion

*Determining the characteristics of Omeprazole*

**Figure 1:**
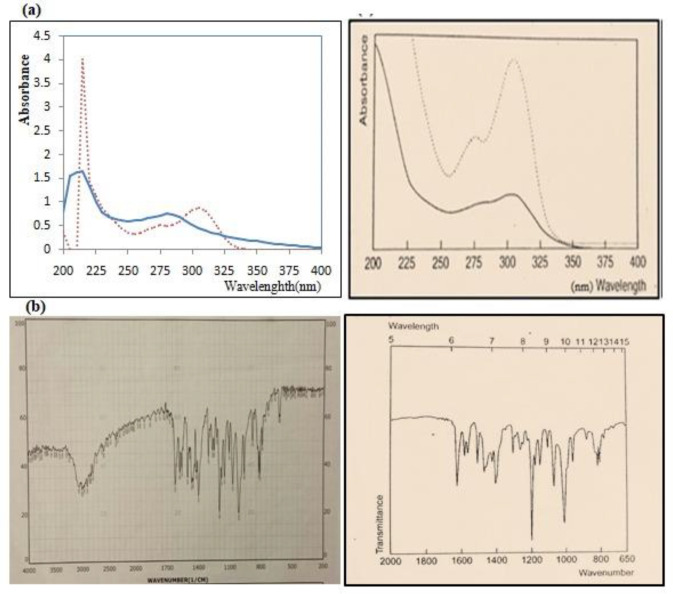
(a) Comparison UV spectrum of purchased Omeprazole (left) with standard Omeprazole in Clark reference (right). The UV spectrum of Omeprazole in a 0.1 M acidic medium (continuous line) and in 0.1 M alkaline medium (dotted point curve), (b) Comparison IR spectrum of purchased Omeprazole (left) with standard Omeprazole in Clark reference (right)

**Figure 2:**
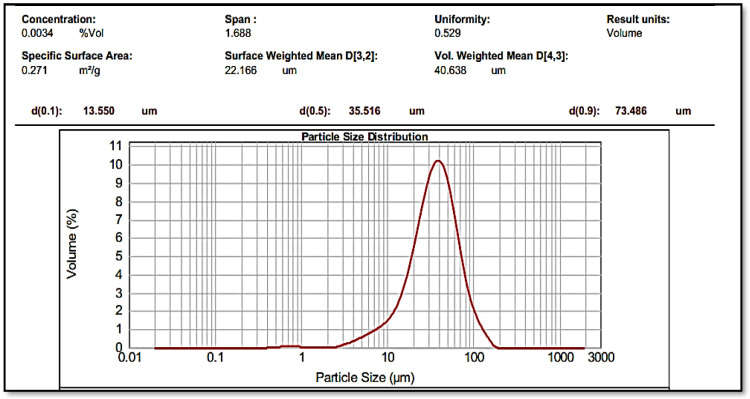
The particle size of thinned and sieved Omeprazole with average particle size of 35.516 µm

After determining the melting point (155 °C), the IR spectrum, the UV spectrum and matching them with values mentioned in Clark reference, size of the Omeprazole particles was reduced before the suspension was made (Figure 1). The final average size for powder particles was 35.516 μm showing a good uniform size (Figure 2). From the pharmaceutical perspective, products containing particles larger than 0.1 µm are known as suspensions, but for common suspensions, the particle size is between 1-50 µm (28). Therefore, this powder was used for further studies. 

*Preparation of Omeprazole suspension*

Based on the adsorption assays of S-layer protein extracted from *L. acidophilus* ATCC 4356 through the QCM method, in the previous study, the content of adsorbed S-layer protein on the gold electrode was 2941.7 × 10^-8^ kg/m^2^ and the amounts of adsorption mass on the silicon dioxide electrode were 1845.6 × 10^-8 ^kg/m^2 ^(22). This process was done in order to calculate the amount of S-layer protein needed to cover the whole surface of the drug. The average content of adsorbed S-layer protein on the electrode surfaces of the QCM device was calculated equaled to 2500 × 10^-8^ kg/m^2^. According to the results of the analysis done on the Omeprazole powder size, the specific surface area was reported as 0.271 m^2^/g (Figure 2). In this way, the amount of S-layer protein required to completely cover 5 mg of drug was estimated to be about 35 µg. 

*Detection of drug coating by determining changes in protein concentration*

Computing the amount of S-layer protein used to cover the drug through Bradford method is very simple and cost-effective. Despite the great efforts made in this study, this method did not provide acceptable results. Ideally in the Bradford method, pure proteins such as Bovine Serum Albumin and Globulin are used as standard proteins to draw a standard curve. The difference between the nature of the S-layer protein and Serum Albumin protein used to draw the standard Bradford curve leads to determine the protein values ​​relatively. In addition, the unrealistic values for S-layer proteins before and after the coating process can be due to the equilibrium of reaction in the binding of S-layer monomers to the surface. The lack of repeatability of Bradford’s results in coating experiments has led to the removal of this method. In the following, the stability of coated Omeprazole was investigated through HPLC method to evaluate the efficacy of S-layer protein in the coating of the drug. 

*HPLC method for Omeprazole assay*

**Figure 3:**
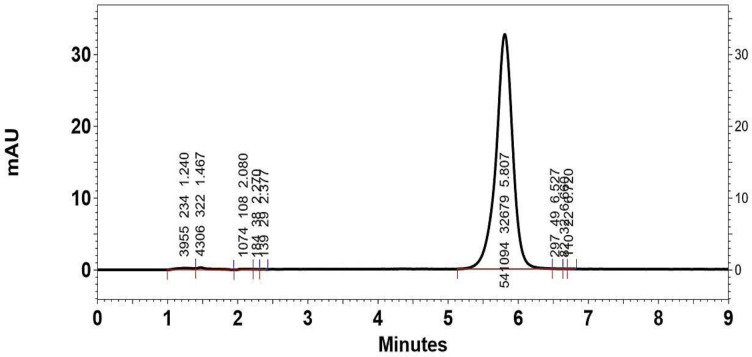
Typical HPLC chromatograph of Omeprazole in mobile phase with retention time of 5.807 min. The y-axis of the chromatograph is a measurement of the intensity of adsorbance (in units of mAU, or milli-Adsorbance Units). The x-axis is in units of time (typically minutes), and is used to determine the retention time for each peak

Figure 3 shows a typical chromatograph for the intact drug dissolved in mobile phase with 10 µg/mL concentration (retention time = 5.807 min). The HPLC method adopted in this article enabled to achieve satisfactory quantitative analysis of Omeprazole within the selected concentration range. Standard curves were linear having correlation coefficient of 0.9998 ± 0.00052. The intra-day and inter-day precision of the method has been shown in Table 2. The method was found to be precise as the intraday relative standard deviation percent (RSD %) of three replicate determinations for one day at the concentration in the range of 0.35 to 1.48 % and the inter-day precision ranged from 2.22 to 8.08 % (Table 2). 

*Evaluation of different methods for Omeprazole coating with S-layer protein*

*Coating the drug during the dialysis process*

**Figure 4:**
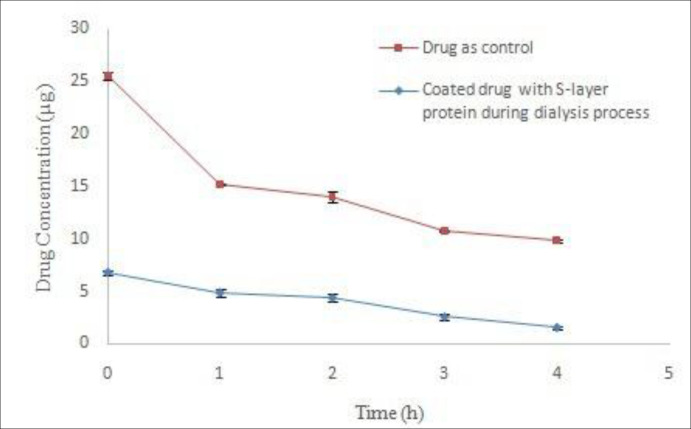
Comparison of Omeprazole stability coated with S-layer protein of L.acidophilus ATCC4356 during dialysis with stability of control drug in acetate buffer (pH 5).

Adding the drug to the dialysis bag was done during the early hours of dialysis. The results of stability studies on coated drug during the dialysis process in acetate buffer (pH 5) indicated the instability of drug compared to control drug (Figure 4). Nonlinear regression analysis revealed the significant difference in Y intercept between the coated drug and the control drug (*P *˂ 0.05) during the dialysis process. Moreover, comparison of decomposition rate of the samples with nonlinear regression does not show any significant difference (*P *˃ 0.05). The lack of drug coatings during dialysis is probably due to the greater tendency of S-layer protein subunits to interconnect with each other rather than to bond to the surface of the drug particles when the extraction agent exits from the dialysis environment. In addition, the presence of very low amounts of GuHCl in the early hours of dialysis led to drug instability compared with control drug. Therefore, this method is not suitable for coating the drug. 

*Coating the drug using S-layer protein monomers resulting from dialysis process*

**Figure 5:**
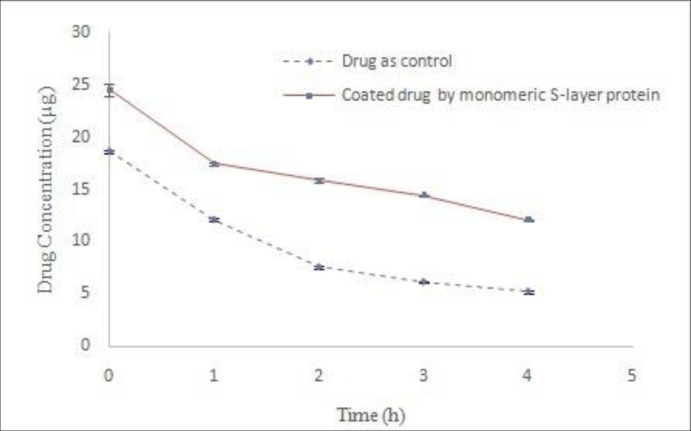
Comparison of Omeprazole stability coated with monomeric S-layer protein of L.acidophilus ATCC4356 with stability of control drug in acetate buffer (pH 5), (Mean ± SD, n: 3).

The solution obtained from the S-layer protein extraction at the end of the dialysis process is completely opaque and milky due to the self-assembling of S-layer monomeric units as well as the formation of polymeric plates. To attain monomeric units, extracted protein was centrifuged (61000 ×g, 30 min, 4 °C) after dialysis process (18). Transparent clear solution containing protein monomers was used for drug coating studies. The results drug stability in acetate buffer (pH 5) indicated that the use of S-layer protein monomers have a positive effect on drug coating (Figure 5). Significant differences of Y intercept between both control and treatment samples were revealed by nonlinear regression analysis (*P *< 0.05). This difference can indicate the protective effect of S-layer protein on the drug during the coating process. Additionally, the comparison of the line slope difference between both control and treatment samples by nonlinear regression analysis and Boolean algebra showed a significant difference (*P *< 0.05) and this difference was reported as 2.223. In other words, the coating of drug by the S-layer protein decreased the rate of drug decomposition up to 2.223. Furthermore, other studies indicate that monomeric units of S-layer protein are required for surface coatings. Hollman *et al. *used clear monomeric solution of S-layer of *L. brevis* and *L. kefir* bacteria for liposome coating, too (9, 11). Ucisik *et al.* also utilized clear monomeric solution of S-layer protein of *Geobacillus stearothermophilus (G. stearothermophilus) *to cover the surface of emulsan (29, 30). Varg *et al.* used S-layer clear solution after centrifugation of the dialysis product for immobilization of S-layer of *Lysinibacillus sphaericus* on alginate matrix (3).

*Optimization of Omeprazole coating by S-layer protein*

As discussed in the previous section, the use of S-layer protein monomers extracted from the dialysis process is a suitable method for coating particles of the drug. Various factors influence the process of drug coating. Initially, according to previous studies, a number of influential factors including temperature, time, shaking speed and proper ratio of S-layer protein to drug surface were selected (22). The process of drug coating by monomeric units of S-layer was done in optimum conditions. In order to evaluate the reproducibility, the process of drug coating was repeated for 6 times in the optimum conditions and the CV was calculated as percentage (Table 3). The CV was found to be about 5%, which indicates the repeatability of the work. In fact, this value of CV indicates the high accuracy of adopted method. After verifying the repeatability of the work, the effects of other various factors on drug coating were evaluated using S-layer protein by one factor at a time method in 3 replications. 

*The effect of time in the drug coating process using S-layer protein*

**Figure 6.**
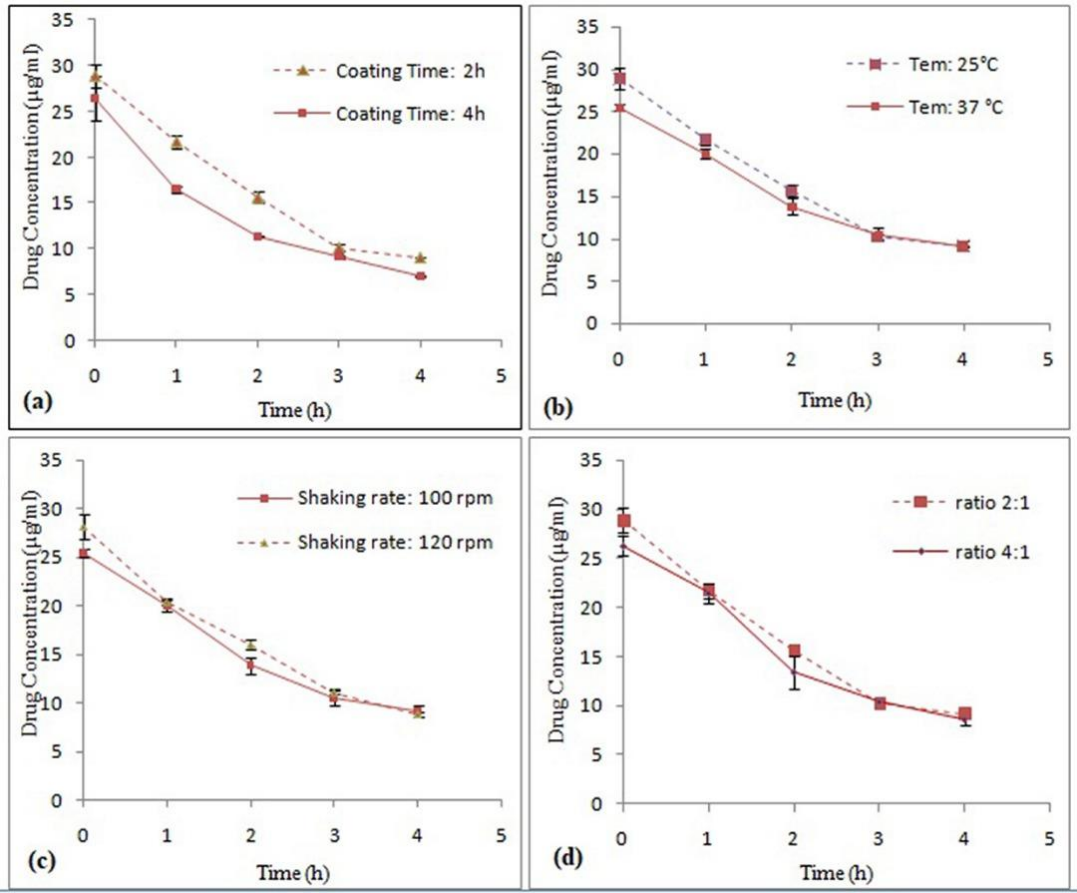
Comparison of effect different factors ((a)Time, (b)Temperature, (c) Shaking rate and (d) S-layer protein amount/Omeprazole Surface ratio) on Omeprazole stability coated with S-layer protein of L. acidophilus ATCC4356 in acetate buffer (pH 5), (Mean ± SD, n: 3).

In order to select the appropriate time range, 2-hour and 4-hour intervals were used. The results showed that the maximum attachment of S-layer proteins to drug particles was occurred within 2 h (Figure 6a). Nonlinear regression analysis revealed the significance of the difference in Y intercept in both time intervals (*P *< 0.05). This difference can indicate the protective effect of S-layer protein on the drug during the coating process. But comparison of decomposition rate of two samples with nonlinear regression does not show any significant difference (*P *˃ 0.05). Probably, this process is beneficial for the coating of the drug by the S-layer protein in the early hours, but by elapsing the time, the removal of protein from the surface of the drug occurs. Hence, the time of 2 h was chosen as the optimal time. Eslami *et al. *also reported the optimal time of 2 h for coating *Lactobacillus casei (L. casei*) probiotic bacteria using S-layer of *L. acidophilus* ATCC 4356 (22). Bingle *et al.* investigated the reattachment *of Aztobacter vindandii *S-layer proteins onto the cell wall of the desired bacteria whose S-layer was isolated. They showed that the maximum reattachment of S-layer proteins to the cell wall of the bacteria occurred within 2 h at 30 °C (31).

*The effect of temperature in the drug coating process using S-layer protein*

With respect to maximum Omeprazole stability temperature (40 °C), temperatures of 25 °C and 37 °C were selected to examine the coating process. In this study, the temperature of 25 °C was selected as the optimum temperature for drug coating process using S-layer protein monomers. The results showed that the increase in the temperature of the coating process did not have an effect on increasing the Omeprazole stability (Figure 6b). Nonlinear regression analysis revealed the significance of the difference in Y intercept in both temperatures (*P *< 0.05) while comparison of decomposition rate of two samples with nonlinear regression did not show any significant difference (*P *˃ 0.05). Probably due to the equilibrium of the binding of S-layer monomers to the surface reaction, the equilibrium has led to separation of some of the adsorbed S-layer proteins from the drug surface as the temperature increased. In the studies conducted by Hollmann *et al. *self-assembling effects on positively charged liposomes by S-layer proteins of *L. kefir* and *L. brevis* bacteria were examined. During this research, the liposome coating process was performed at 25 °C (10, 11). However, in the studies done by Lund *et al. *it was determined that the maximum binding of S-layer proteins extracted from *Aeromonase salmonicida* to a variety of bacteria of this family occurred at 30 °C and 37 °C during 30 min (32). The difference in temperature and time in these studies is probably due to the different nature of the S-layer proteins. 

*The effect of shaking rate in the drug coating process using S-layer protein*

Since the drug particles are fine, the particles were completely deposited in the sedentary state and adhesion of drug particles to the walls of the container occurred by increasing the speed of the shaker. Through quality control of the process, the speeds of 100 and 120 rpm for the shaker were selected to examine the effect of this factor on Omeprazole coating process. The results showed that increasing the speed of the shaker did not have a significant effect on increasing the drug coating and hence increasing the drug stability (Figure 6c). Nonlinear regression analysis revealed the significance of the difference in Y intercept in both shaking rate (*P *< 0.05) while comparison of decomposition rate did not show any significant difference (*P *˃ 0.05). This may be due to the fact that increasing movement of protein biomolecules at higher shaking rates causes detachment of the protein molecules from the drug surface. In different studies done on adsorption of S-layer protein on different surfaces, various shaker speeds have been reported because of the various natures of surfaces. For example, Eslami *et al.* reported an optimum rpm of 50 for coating *L.casei* probiotic bacteria using S-layer protein of *L. acidophilus *ATCC 4356 because the survival rate of *L.casei *cells was significantly reduced in higher speeds of shaker (22).

*Selection of suitable ratio of S-layer protein amount/Omeprazole Surface in the drug coating process using S-layer protein*

For one factor at a time experiments, ratios of 2:1 and 4:1were selected. The results showed that the ratio of 2:1, which is about 70 µg of S-layer protein for 5 mg of drug is preferable (Figure 6d). Nonlinear regression analysis revealed the significant difference in Y intercept in two ratio (*P *< 0.05), but comparison of decomposition rates of both samples did not show any significant difference (*P *˃ 0.05). No significant change in the adsorption of protein on the surface of the drug particles by increasing the amount of S-layer protein indicates that coating of drug has been completed in the ratio of 2:1. In other words, in this ratio, the entire surface of the drug has been covered by S-layer molecules and additional S-layer molecules in the medium are reluctant to bind to S-layer molecules on the surface of the drug. In studies conducted by Hollmann *et al. *the effect of self-assembling was examined on positively charge of liposomes by different concentration of S-layer proteins of *L. kefir* and *L. brevis* bacteria. Optimal concentrations of 45 μg/mL and 200 μg/mL were reported for S-layer protein of *L. kefir* and *L. brevis*, respectively (11). 

*Effect of different concentrations of EDTA to prevent the self-assembling process of S-layer proteins*

**Figure 7:**
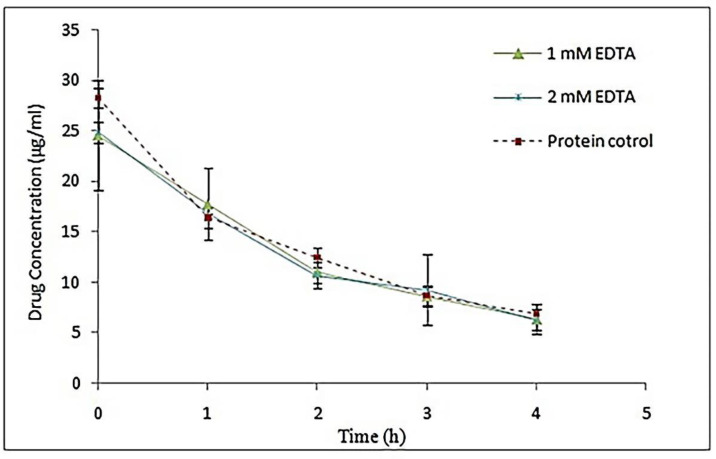
Comparison of Omeprazole stability coated with S-layer of L. acidophilus ATCC4356 in the presence of different concentrations of EDTA with stability of control drug coated with EDTA-free S-layer protein in acetate buffer (pH 5), (Mean ± SD, n: 3).

One of the ways to avoid the self-assembling process of S-layer proteins is to use EDTA (33). In this study, concentrations of 1 and 2 mM of EDTA were utilized in dialysis buffer and protein samples were used to coat the drug and then the stability studies were performed as mentioned before. The results of stability studies have been shown in Figure 7. Non-linear regression analysis showed that the difference in Y intercept in both concentration of EDTA was not significant compared to the control sample (*P *˃ 0.05). Also, there was no significant difference between the line slopes in both treatment sample and control sample (*P *˃ 0.05). Thus, the use of EDTA in different concentrations did not have any effect on the efficiency of the extracted S-layer protein monomer. In some studies, EDTA has been used with different concentrations to keep protein monomers stable for longer periods of time. Teixeira *et al. *added 1 mM EDTA to dialysis buffer for kinetic studies of S-layer protein. Their results indicated that the S-layer protein monomer solution remained stable for 2 months in a refrigerator at 4 °C, and small protein assemblies did not appear (33). However, Breitwieser *et al.* used EDTA with a concentration of 2 mM in their dialysis buffer. They reported that despite EDTA, protein monomers were not completely stable and there was a tendency to form oligomeric units (2).

*The hydrophobicity changes of the drug surface with inoculation in 0.2% sodium taurocholate*

**Figure 8:**
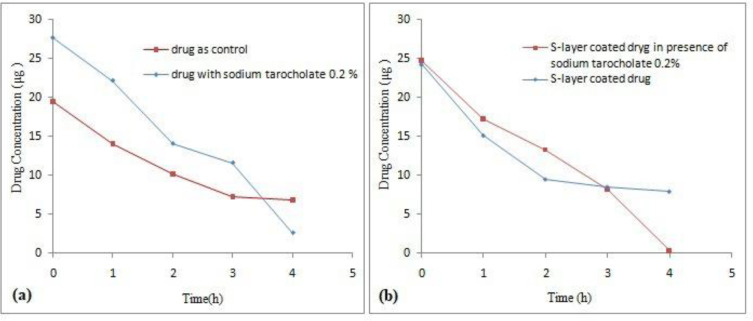
The effect of 0.2% sodium taurocholate on (a) stability of the drug and (b) stability of Omeprazole coated with S-layer of L. acidophilus ATCC4356 in the presence in 0.2% sodium taurocholate in acetate buffer (pH 5), (Mean ± SD, n: 3).

One idea in current study was to change the hydrophobicity of the drug surface and then to investigate the effect of this change on how they are bonded to the S-layer protein (22). The drug was inoculated with 0.2% sodium taurocholate solution in order to evaluate the effect of 0.2% sodium taurocholate on the drug. Nonlinear regression analysis revealed a significant difference between Y intercept of both control samples and 0.2% sodium taurocholate (*P *˂ 0.05), but there was no significant difference between the line slope between treatment sample and control sample (*P *˃ 0.05). As shown in Figure 8a, a sharp drop in concentration of the drug occurred from the third hour. In Figure 8b, the S-layer coated drug in the presence of sodium taurocholate has been exposed to a sharp drop in concentration from the third hour. In fact, 0.2% sodium taurocholate initially showed a protective effect on the drug, but as the time elapsed, it had a destructive effect on the drug. Nonlinear regression analysis also showed that the difference between Y intercept and the line slope in the both control and coated drug in the presence of sodium taurocholate samples were not significant (*P *˃ 0.05). While in this study, interfering effect between sodium taurocholate and Omeprazole was observed, in a study conducted by Eslami *et al. *use of 0.2% sodium taurocholate increased the surface hydrophobicity of *L.casei* bacteria and had a positive effect on the coating of *L. casei* bacteria using *L .acidophilus* ATCC 4356 S-layer protein (22).

*Effect of various sugars on the Omeprazole coating process*

**Figure 9:**
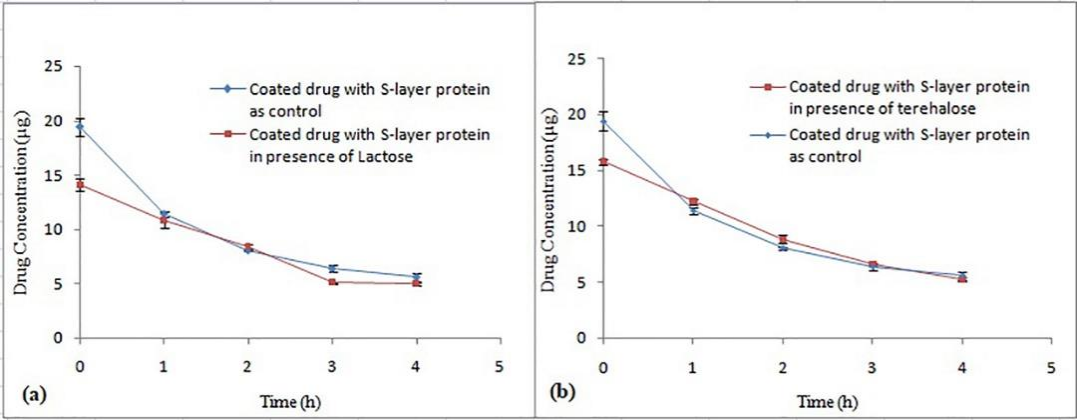
The effect of different sugars, (a) Lactose and (b) Trehalose on stability of Omeprazole coated with S-layer of L. acidophilus ATCC4356 in acetate buffer (pH 5), (Mean ± SD, n = 3).

Theoretically, sugar is expected to function as a binding agent between drug particles and S-layer proteins by having a sugar-based functional group. Consequently, concentration of 5% for lactose and trehalose sugars were used (Figure 9). In both control and treatment samples for two sugars, the difference in Y intercept was significant (*P *< 0.05), but comparison of decomposition rate of two samples does not show any significant difference (*P *˃ 0.05). None of the sugars have affected the improvement of the drug coating process and thus increase of drug stability in acidic pH. Eslami *et al. *used lactose solution at concentrations of 2% and 5% and they reported that the use of 5% lactose had a positive influence on improving the viability of *L.casei* bacteria coated with *L. acidophilus* ATCC 4356 S-layer protein, after inoculation in the simulated gastric medium, while 2% lactose did not show such effect (22).

*Coated drug by dry S-layer protein*

**Figure 10:**
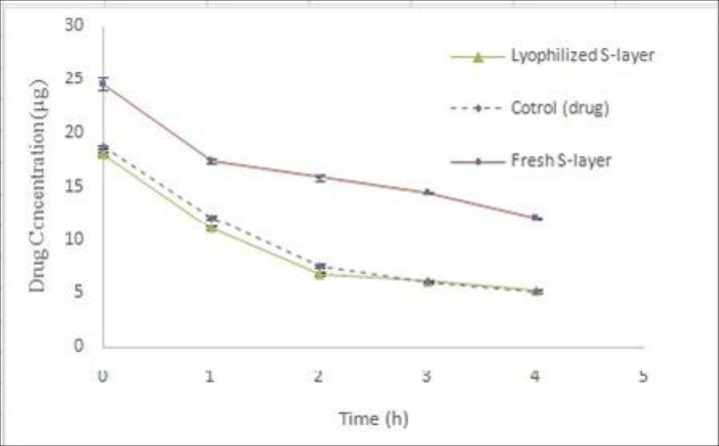
Comparison of Omeprazole stability using dry S-layer protein of L.acidophilus ATCC4356 with Omeprazole stability using freshly extracted S-layer protein in acetate buffer (pH 5), (Mean ± SD, n: 3).

Considering that the basis of present work was to use surface monomeric units and this involved the extraction of fresh S-layer protein. However, if it is possible to use a dry S-layer protein, it will be cost effective in terms of time and cost. In this regard, the drug coating process was performed using dry S-layer protein in optimal conditions and its results were compared with the freshly extracted protein. The results showed that the coating process of the drug was not well done using the dry S-layer protein (Figure 10). Nonlinear regression analysis showed that the difference of Y intercept between coated drug using dry S-layer protein and coated drug using fresh S-layer protein was significant (*P *˂ 0.05), but comparison of decomposition rate of two samples does not show any significance difference (*P *˃ 0.05). In fact, the dry S-layer protein compared to the freshly extracted S-layer protein did not have a better effect on improving the coating of the drug and thus not increasing its stability. At the time of preparing the appropriate concentration of dry S-protein for the coating process, the resulting solution is not completely transparent and in fact the dry protein is not completely soluble and most of the remaining parts of it remain polymeric. Because of the need for S-layer monomers to coat the drug, the resulting dry S-layer protein solution during the coating process did not have tendency to attach on the particles of the drug. In the studies done by Hollmann *et al.* on coating of liposome surface using S-layer proteins of *L. kefir* and *L. brevis* bacteria, fresh extracted solution of S-layer proteins was immediately used (10, 11). Ucisik *et al. *used a clear monomeric solution of S-layer protein of *G. stearothermophilus* to cover the surface of emulsan (30).

**Table 1:** Parameters and variables used in one factor at a time experiments

**Parameters**	**variables**
Time	2,4 h
Temperature	25, 37 °C
Shaking rate	100,120 rpm
S-layer protein amount/Omeprazole Surface	2/1, 4/1
EDTA	1, 2 mM
Taurocholate sodium	0.2%
Sugars (5%)	Lactose, trehalose

**Table 2:** Inter- and intra-day precision (n = 3).

**Concentration (µg/mL)**	**RSD (%)**	
	**Intraday**	**Interday**
10	1.48	2.22
12.5	0.35	3.03
25	0.88	8.08
50	1.17	2.66
75	0.77	5.50
100	0.42	5.68

**Table 3:** Determination of drug concentration at different times to assess the stability of Omeprazole coated with monomeric S-layer protein of L. acidophilus ATCC4356 in acetate buffer (pH 5), (Mean ± SD, n: 6)

**Time (h)**	**Coated drug concentration ** **(µg/mL)**	**SD**	**CV (%)**
0	25.1564	1.319555	5.245404
1	16.34433	0.853637	5.222829
2	12.09534	0.863281	7.1373
3	9.238918	0.517674	5.603187
4	7.391818	0.489736	6.625374

## Conclusion

The protein subunits of the S-layer have been attached to each other through non-covalent bonds and detached into monomeric units by chaotropic agents such as GuHCL. Due to self-assembly ability of S-layer proteins at different surfaces, use of this type of protein monomers of S-layer to coat suspension of omeprazole has increased the stability of the drug in acidic conditions. Although the optimization of various factors did not significantly affect the stability of the coated drug in acidic conditions, the decrease of decomposition rate of coated omeprazole by lactobacillus S-layer protein can lead to use of this kind of proteins to increase the stability of the other unstable gastrointestinal drugs in gastrointestinal system.
